# The transmission risk of multidrug-resistant organisms between hospital patients and their pets – a case−control study, Germany, 2019 to 2022

**DOI:** 10.2807/1560-7917.ES.2024.29.39.2300714

**Published:** 2024-09-26

**Authors:** Carolin Hackmann, Antonia Genath, Désirée Gruhl, Anna Weber, Friederike Maechler, Axel Kola, Frank Schwab, Stefan Schwarz, Antina Lübke-Becker, Thomas Schneider, Petra Gastmeier, Rasmus Leistner

**Affiliations:** 1 Institute of Hygiene and Environmental Medicine, Charité – Universitätsmedizin Berlin, Berlin, Germany; 2 Institute of Microbiology and Epizootics, School of Veterinary Medicine, Freie Universität Berlin, Berlin, Germany; 3 Veterinary Centre of Resistance Research (TZR), School of Veterinary Medicine, Freie Universität Berlin, Berlin, Germany; 4 Division of Gastroenterology, Infectious Diseases and Rheumatology, Medical Department, Charité – Universitätsmedizin Berlin, Berlin, Germany

**Keywords:** one health, antimicrobial resistance, pet ownership, whole genome sequencing

## Abstract

**Background:**

Carriage of multidrug-resistant organisms (MDROs) in humans constitutes an important public health concern. Cross-transmission of bacteria between animals and humans has been demonstrated before.

**Aim:**

Our aim was to quantify the risk factor ‘pet ownership’ for MDRO colonisation in hospital patients.

**Methods:**

We performed a matched case–control study from 2019 to 2022 in Berlin, Germany and compared MDRO-positive and MDRO-negative patients in terms of contact with pets and other risk factors for MDRO acquisition. Patients completed a questionnaire-based interview and provided nasal and rectal swabs. Pet owners provided swab samples from the throat and stool of their pets (dogs and cats). Phenotypically matching samples of owners and pets were analysed via whole genome sequencing.

**Results:**

The analyses included 2,891 patients. Reported pet ownership was 17.7% in MDRO-positives (154/871) and 23.4% in MDRO-negatives (472/2,020). Among 397 owner–pet pairs, we identified one pair sharing genotypically indistinguishable pathogens (0.3%). A risk factor analysis of pet ownership was performed for carriers of meticillin-resistant *Staphylococcus aureus* (MRSA) (OR = 0.662; 95% CI: 0.343–1.277), vancomycin-resistant enterococci (VRE) (OR = 0.764; 95% CI: 0.522–1.118) and multidrug-resistant Gram-negative bacteria (MDR-GNB) (OR = 0.819; 95% CI: 0.620–1.082). Colonisation with MDRO was rare in pets, and dogs were more often colonised than cats (MRSA: 0% vs 0%, VRE: 1.5% vs 1.0%, MDR-GNB: 17.2% vs 3.6%).

**Conclusion:**

Transmission of MDROs between humans and pets is possible though rare. In an urban living space, neither cat nor dog ownership appears as a relevant risk factor for MDRO carriage in hospital patients.

**Key public health message**

**What did you want to address in this study and why?**

Pets and owners share the same environment and live in close contact. Colonisation, which is a symptom-free existence of certain bacteria for example in the gut or on the skin, with multidrug-resistant organisms (MDRO) can occur in humans as well as in pets. We wanted to examine if pet ownership is a risk factor for MDRO colonisation in hospital patients.

**What have we learnt from this study?**

Examining 2,891 patients, we did not identify pet ownership as a risk factor for colonisation with the most common MDROs, namely meticillin-resistant *Staphylococcus aureus* (MRSA), vancomycin-resistant enterococci (VRE) and multidrug-resistant Gram-negative bacteria (MDR-GNB). Direct transmission between pet and owner was rare and occurred only once among 397 owner–pet pairs.

**What are the implications of your findings for public health?**

This evidence can be used to answer the question whether pet ownership should be added to the list of risk factors for MDRO colonisation. It would help identify patients with an increased risk of colonisation at an early stage. Especially regarding antimicrobial resistance, the One Health approach, which considers the health of humans, animals and the environment as closely related, is of high relevance to develop overarching strategies.

## Introduction

Multidrug-resistant organisms (MDROs) are one of the major public health concerns of our time [1]. In a recent global burden of disease study, Murray et al. estimated 1.27 million deaths directly attributable to resistant organisms globally in 2019 [2]. Six pathogens are responsible for most of these antimicrobial resistance (AMR)-associated deaths, among them *Escherichia coli*, *Staphylococcus aureus*, *Klebsiella pneumoniae* and *Pseudomonas aeruginosa* [2]. These four pathogens also belong to the most relevant MDROs associated with nosocomial infections, namely meticillin-resistant *S. aureus* (MRSA), third-generation cephalosporin-resistant (3GCR) as well as carbapenem-resistant (CR) Gram-negative bacteria (GNB), grouped under the term multidrug-resistant Gram-negative bacteria (MDR-GNB). The third group of highly relevant MDROs in the hospital are vancomycin-resistant enterococci (VRE). Apart from nosocomial infections due to transmission in the hospital, infections can also originate from colonisation before hospitalisation. Here, the interface between humans, animals and the ecosystem is of great importance in terms of the One Health concept [3,4]. To develop preventive measures such as specific risk factor-adjusted screening programmes, it is necessary to be aware of risk factors for MDRO acquisition before hospitalisation.

Humans and animals often share the same environment and therefore also share communicable diseases [5]. Thus, MDRO colonisation and infection can also occur in wild animals, livestock and pet animals such as cats and dogs [6,7]. Colonisation with MDRO in pets and owners from the same household as well as transmission between them has been reported earlier [8-11]. However, it is still unclear if pet ownership is associated with an overall higher risk of MDRO acquisition. While veterinarians do have a higher risk of MDRO colonisation [12], a recent systematic meta-analysis could not confirm a generally higher risk for pet owners [13]. We therefore performed a case–control study to assess pet ownership as a risk factor for MDRO carriage in hospital patients and to estimate the rate of transmission between pets and owners.

## Methods

### Study design

#### Study period and population

We performed a case–control study at the Charité University Hospital in Berlin, Germany from June 2019 to September 2022. Cases were defined as patients who tested positive for MDROs (used hereafter for: MRSA, VRE and MDR-GNB) in swab samples from nose and rectum within 72 h after hospital admission. Controls were defined as patients who tested negative for MDROs during the first 72 h of their hospital stay.

Participants were eligible for recruitment if they were 18 years or older. Accompanying persons, patients under legal guardianship, patients unable to communicate in German or English, or patients not capable of study participation due to poor general health were excluded from recruitment. The reasons for poor general health included acute sickness, chemotherapy or recent surgeries.

Participants were selected from the Charité patient population, swabs were taken by study staff members and were not part of routine surveillance. To detect potential cases, we approached patients who tested positive for MDROs in the 72 h after hospital admission and patients who had been infected or colonised with MDROs in previous hospital stays at the Charité. To detect potential controls, we approached patients who tested negative for MDROs in the 72 h after hospital admission and newly admitted patients during the first 72 h of their hospital stay. We aimed at a case:control ratio of 1:2.

A detailed study protocol is provided in Supplement 1.

#### Data collection

All participants were swabbed by study staff members (nose and rectal swabs) and completed a questionnaire-based interview performed by our study staff members. If the participants owned cats or dogs (further referred to as pets), they were asked to answer further questions regarding their pet’s health status in the past 6 months, diet of their pets, the closeness of contact and behaviour of the pets. We obtained the Charlson comorbidity index (CCI) for each patient based on diagnosed comorbidities [14]. We grouped the original Charlson comorbidity categories in 10 disease categories: heart disease, cerebrovascular disease, neurological disease, lung disease, rheumatic disease, gastrointestinal disease, liver disease, diabetes, renal disease and cancer/immunological disease.

Nasal and rectal swabs of all participants were tested for MRSA, VRE and MDR-GNB.

#### Matching

For the risk factor analysis for each type of resistant organism (MRSA, VRE, MDR-GNB), we performed retrospective matching. We matched each MDRO-positive patient of a specific type with two MDRO-negative patients from the same ward. The MDRO-negative patients from the same ward were chosen randomly and multiple use of MDRO-negative controls was possible. Cases were excluded if pairing with two adequate controls was not possible. Sensitivity analyses were performed to assess the influence of no matching at all and matching with single use of controls on the results. For the analysis of pet samples and the calculation of transmission rate between pets and humans, we included the whole study cohort.

#### Animal screening

All study participants who were owners of cats or dogs (further referred to as pet owners) received screening kits to test their pets at home. Pet owners collected swab samples of their pet’s throat and stool. In addition, they answered a short questionnaire to provide information on the health status of each pet at the time of sampling. When pathogens isolated from human and pet samples from the same household shared the same type of genus and species, we performed whole genome sequencing (WGS) to examine the genetic relatedness.

### Microbiological methods

Nasal swab samples from humans and throat swab samples from pets were inoculated onto chromogenic MRSA agar (ChromID MRSA, bioMérieux, Marcy-l’Étoile, France). For further investigation of MRSA-positive specimens, we performed latex agglutination tests detecting clumping factor (Staphaurex Plus, Remel, Lenexa, United States (US)) and penicillin-binding protein (PBP2 test kit, Oxoid, Wesel, Germany). Rectal swab and stool samples were inoculated onto chromogenic agar (ChromID ESBL-Agar, bioMérieux) and McConkey agar plates (McConkey-Agar, bioMérieux). Identification of MDR-GNB species and antimicrobial susceptibility testing were performed with the Vitek2 System (bioMérieux, Vitek AST-panel N223 for MDR-GNB with EUCAST-breakpoints for both humans and animals). We determined ESBL positivity by comparing the combination of cefpodoxime and clavulanic acid with cefpodoxime alone within the AST N223 panel. Production of AmpC was not included with ESBL positivity. For the identification of VRE, we used ChromID VRE agar plates (bioMérieux) and disc diffusion method with vancomycin and teicoplanin (Mast Group Ltd., Bottle, United Kingdom). Identification of species and antimicrobial susceptibility testing were performed with the Vitek2 System. All MDROs were stored at −80 °C for further sequence analysis. Only samples showing phenotypically matching pathogens were sequenced. After culturing on blood agar, we extracted DNA using UltraClean Microbial DNA isolation kit (Qiagen, Hilden, Germany). Short read sequencing was performed on the MiSeq system (Illumina Inc., San Diego, US). For cgMLST analysis, quality trimming, de novo assembly and gene-by-gene comparison were carried out with the SeqSphere+ software (Ridom GmbH, Germany, version 4.1.9 at default settings) using the published *E. coli* and *Enterococcus faecium* cgMLST task templates [15,16]. We determined cgMLST clusters using pairwise allelic differences between isolates with a transmission cut-off of ≤ 10 alleles difference for *E. coli* and ≤ 20 alleles for *E. faecium*, which represents default settings.

### Statistical methods

In the descriptive analysis, number and percentage or median and interquartile range (IQR) were calculated, depending on the distribution of the variable. Differences were tested using Pearson's chi-square test, Fisher's exact test or Wilcoxon rank sum test. We performed a univariable analysis and a multivariable analysis to estimate the independent effect of variables on the risk of MDRO acquisition. We calculated odds ratios (OR) and their 95% confidence intervals (CI) based on a conditional logistic regression for each MDRO type. No OR was calculated when there were no events of a variable in one or two of the observed groups. Multivariable models were based on directed acyclic graphs (DAG) using the browser-based version of the software DAGitty [17]. For multivariable analyses, DAGs were constructed. Adjustable variables were selected based on their relationship to the exposure (pet ownership) and the outcome (MRSA, VRE or MDR-GNB colonisation) in DAGs for each type of MDRO. The selected adjustment sets for the three separate analyses can be found in Supplementary Figure S6. The relationship between variables, outcome and/or exposure was estimated based on literature findings. All models were adjusted for age and sex. All statistical analyses were performed using R, version 4.2.1 [18]. A value of p < 0.05 was considered significant. Due to the expected low number of CR-GNB-positive patients and the concordance of bacterial species with 3GCR-GNB species, we analysed 3GCR-GNB- and CR-GNB-positive patients as one group in all analyses.

## Results

### Study population

**Figure 1:**
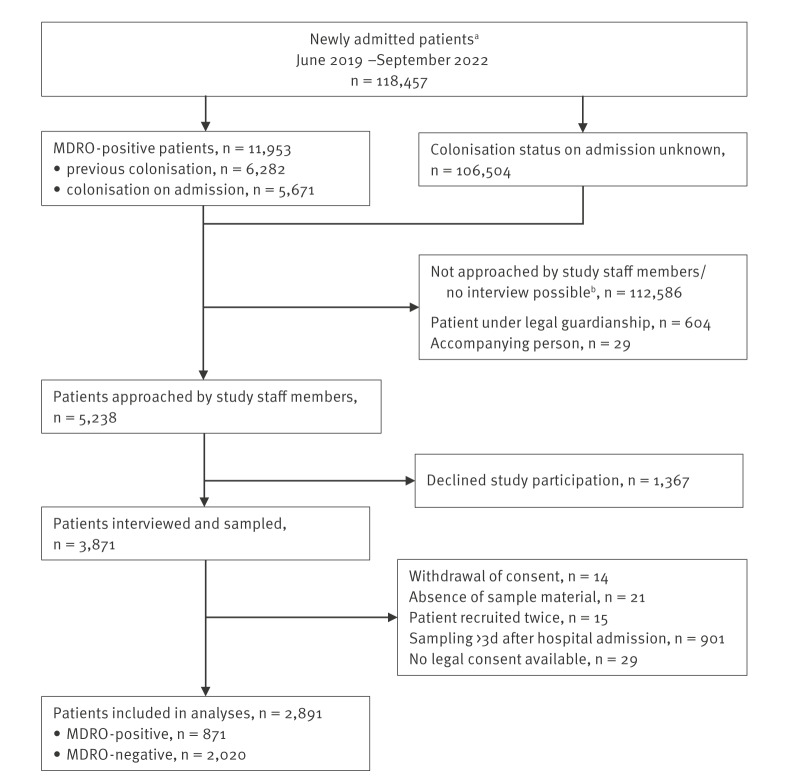
Recruitment of patients, study on transmission risk of multidrug-resistant organisms between hospital patients and their pets, Charité University Hospital, Germany, June 2019–September 2022 (n = 118,457)

In total, we included 2,891 participants in our analyses. Among those, 871 (30.1%) tested positive for at least one type of MDRO and were therefore defined as cases. Patients who tested negative for all three types of MDRO were defined as controls (n = 2,020; 69.9%). The flowchart for the recruitment process is shown in Figure 1.

d: days; MDRO: multidrug-resistant organisms.

^a^ Minimum age 18 years, no unidentified persons, no accompanying persons, no readmissions of the same person.

^b^ Patients not capable of study participation due to poor general health (e.g. acute sickness, chemotherapy or recent surgeries) or language barrier.

All patients were queried regarding demographic data, known risk factors for MDRO acquisition and their contact with pets. Furthermore, we obtained the CCI for each patient to assess underlying comorbidities. The full table of the distribution of characteristic attributes for all included patients, as well as for the groups of MDRO-positives and MDRO-negatives can be found in Supplementary Table S1. An excerpt of demographic and pet-related characteristics is shown in Table 1.

**Table 1:** Distribution of selected characteristics in the study cohort, study on transmission risk of multidrug-resistant organisms between patients and their pets, Charité University Hospital, Germany, June 2019–September 2022 (n = 2,891)

Characteristic	N^a^	Overall n = 2,891		MDRO-positive n = 871		MDRO-negative n = 2,020	
		n	%	n	%	n	%
Sex (male)^b^	2,891	1,608	55.6	507	58.2	1,101	54.5
Median age in years (IQR)	2,891	63 (51–73)		65 (53–74)		61 (50–72)	
BMI in kg/m^2^ (IQR)	2,891	24.8 (22.0–28.7)		24.5 (21.6–28.3)		25.1 (22.2–29.0)	
CCI (IQR)	2,876	4 (2–7)		4 (2–7)		4 (2–6)	
Pet ownership^c^	2,890	626	21.7	154	17.7	472	23.4
Dog ownership	2,890	360	12.5	93	10.7	267	13.2
Cat ownership	2,890	333	11.5	80	9.2	253	12.5
Ownership of small animals other than cats or dogs	2,890	115	4.0	36	4.1	79	3.9
Professional contact with livestock or pets	2,888	163	5.6	43	4.9	120	5.9

BMI: body mass index; CCI: Charlson comorbidity index; IQR: interquartile range; MDRO: multidrug-resistant organisms.

^a^ Available datasets for each characteristic.

^b^ Analysed as a binary variable (male/not male).

^c^ Pets = dogs and/or cats. One patient is excluded here because the answer about pet ownership was inconclusive.

Most of the MDRO-positive participants tested positive for one type of MDRO each (n = 748; 85.9%) while 123 (14.1%) patients carried more than one resistant pathogen type (range: 2–4 different MDRO species). Among the whole study group, 118 (4.1%) patients carried MRSA, 275 (9.5%) carried VRE, 522 (18.1%) carried 3GCR-GNB and 18 (0.6%) carried CR-GNB.

Overall, 985 isolates were obtained from 871 patients (1.1 per colonised patient). Of 277 VRE isolates, 273 belonged to the species *E. faecium* and four isolates to the species *Enterococcus faecalis*. Among 590 isolates of Gram-negative bacteria, the majority were *E. coli* (n = 411), *K. pneumoniae* (n = 102), *Enterobacter cloacae *complex (n = 18) and *P. aeruginosa* (n = 9), others are listed in the Supplement. All 590 isolates of Gram-negative bacteria were resistant against third-generation cephalosporins, 491 isolates were ESBL-positive, 416 were fluoroquinolone-resistant and 18 were carbapenem-resistant. The detailed species distribution and resistance patterns are shown in Supplementary Table S2.

### Risk factor analysis

We performed univariable analyses of demographic characteristics, information on pet ownership as well as established risk factors for MRSA, VRE and MDR-GNB carriage based on matched groups of MDRO-positive and MDRO-negative patients. Complete tables of all characteristics can be found in Supplementary Tables S3-S5. Table 2 shows selected results of the univariable analyses including demographic data, information on pet ownership as well as significant risk factors (p < 0.05) for each analysed group.

**Table 2:** Univariable analysis of matched groups of MDRO-positive and MDRO-negative patients, Charité University Hospital, Germany, June 2019–September 2022 (n= 2,297)

Characteristics		N^a^	MDRO-positive		MDRO-negative		OR	95% CI	p value
			n	%	n	%			
MRSA (n = 351)			n = 117		n = 234				
Male		351	73	62.4	139	59.4	1.12	0.72–1.75	0.604
Female		351	44	37.6	94	40.2	0.90	0.57–1.41	0.604
Median age in years (IQR)		351	64 (50–76)		60 (46–73)		1.01	0.10–1.02	0.100
CCI (IQR)		350	5 (2–8)		4 (1–7)		1.04	0.98–1.11	0.167
Living situation	Family/shared flat	351	74	63.2	162	69.2	1	Reference	
	Alone		39	33.3	68	29.1	1.23	0.75–2.01	0.624
	Nursing home		4	3.4	3	1.3	2.75	0.61–12.3	0.790
	Others		0	0.0	1	0.4	NA	NA	NA
Prior hospitalisation		349	79	67.5	128	54.7	1.87	1.31–3.10	0.015
Prior antibiotic use		336	71	60.7	85	36.3	2.60	1.62–4.16	< 0.001
Central venous catheter		351	29	24.8	34	14.5	2.29	1.21–4.32	0.011
Neurological disease		349	8	6.8	4	1.7	4.00	1.21–13.3	0.024
Pet ownership^b^		351	18	15.4	55	23.5	0.58	0.32–1.06	0.075
Professional contact with livestock or pets		350	6	5.2	15	6.4	0.79	0.30–2.08	0.636
VRE (n = 825)			n = 275		n = 550				
Male		825	172	62.5	300	54.5	1.39	1.03–1.88	0.029
Female		825	103	37.5	250	45.5	0.72	0.53–0.97	0.029
Median age in years (IQR)		825	66 (55–73)		62 (53–71)		1.01	1.00–1.02	0.007
CCI (IQR)		823	5 (3–7)		4 (2–7)		1.06	1.02–1.10	0.006
Living situation	Family/shared flat	823	193	70.2	386	70.2	1	Reference	
	Alone		69	25.1	158	28.7	0.85	0.61–1.18	0.326
	Nursing home		9	3.3	5	0.9	3.52	1.18–10.5	0.024
	Others		2	0.7	1	0.2	3.90	0.35–4.30	0.267
Prior hospitalisation		822	241	87.6	368	76.9	3.92	2.53–6.07	< 0.001
Prior antibiotic intake		824	188	68.4	206	37.5	5.11	3.46–7.55	< 0.001
Urinary catheter		824	36	13.1	42	7.6	2.22	1.28–3.85	0.005
Central venous catheter		824	99	36.0	152	27.6	1.62	1.14–2.30	0.007
Diarrhoea		818	109	39.6	141	25.6	2.06	1.49–2.87	< 0.001
Renal disease		825	82	29.8	110	20.0	1.71	1.22–2.40	0.002
Cancer/immune disease		821	148	53.8	293	53.3	1.66	1.20–2.30	0.002
Pet ownership^b^		825	49	17.8	131	23.8	0.69	0.48–1.00	0.050
Professional contact with livestock or pets		822	15	5.5	37	6.7	0.79	0.42–1.49	0.471
MDR-GNB (n = 1,602)			n = 534		n = 1,068				
Male		1,602	301	56.4	560	52.4	1.18	0.95–1.45	0.133
Female		1,602	233	43.6	508	47.6	0.85	0.69–1.05	0.133
Median age in years (IQR)		1,602	65 (53–74)		61 (50–71)		1.01	1.01–1.02	< 0.001
CCI (IQR)		1,592	4 (2–7)		4 (2–6)		1.03	1.00–1.07	0.032
Living situation	Family/shared flat	1,601	382	71.5	768	71.9	1	Reference	
	Alone		142	26.6	292	27.3	0.98	0.78–1.24	0.859
	Nursing home		7	1.3	7	0.7	1.99	0.70–5.68	0.200
	Others		2	0.4	1	< 0.1	3.99	0.36–44.0	0.259
Prior hospitalisation		1,601	372	69.7	628	58.8	1.70	1.34–2.16	< 0.001
Prior antibiotic use		1,601	318	59.6	348	32.6	4.32	3.31–5.64	< 0.001
Travel to Asia		1,601	34	6.4	38	3.6	1.81	1.13–2.88	0.013
Urinary catheter		1,600	80	15.0	70	6.6	2.85	1.96–4.13	< 0.001
Central venous catheter		1,600	148	27.7	222	20.8	1.65	1.25–2.19	< 0.001
Renal disease		1,601	163	30.5	222	20.8	1.68	1.33–2.13	< 0.001
Diabetes mellitus		1,598	88	16.5	166	15.5	1.07	0.80–1.41	0.663
Pet ownership^b^		1,602	99	18.5	248	23.2	0.75	0.57–0.97	0.030
Professional contact with livestock or pets		1,599	26	4.9	59	5.5	0.87	0.54–1.41	0.573

BMI: body mass index; CCI: Charlson comorbidity index; CI:  confidence interval; IQR: interquartile range; MDR-GNB: multidrug-resistant Gram-negative bacteria; MDRO: multidrug-resistant organisms; MRSA: meticillin-resistant *Staphylococcus aureus*; OR: odds ratio; VRE: vancomycin-resistant enterococci.

^a^ Number of datasets included for each characteristic.

^b^ Pets = dogs and/or cats.

Table 3 shows the result of the multivariable analysis based on prospectively modelled DAGs. We identified living situation as a potential confounder for all three types of MDROs and professional contact with livestock and pets as a potential confounder for MRSA and MDR-GNB colonisation. Further risk factors were prior hospitalisation and prior antibiotic use for MRSA, VRE and MDR-GNB, travel to Asia for MDR-GNB, cancerous diseases for VRE and inserted catheters for MRSA [19-31]. To prevent distortion of results, we decided on minimalistic models including the in DAGs-identified confounders only. The DAGs can be found in Supplementary Figure S6. They did not show pet ownership as a statistically significant risk factor for MRSA (OR = 0.66; 95% CI: 0.34–1.28), VRE (OR = 0.76; 95% CI: 0.52–1.12) or MDR-GNB colonisation (OR = 0.82; 95% CI: 0.62–1.08). In Supplementary Table S7, we provide additional sensitivity analyses which calculated models including the most established risk factors for each type of MDRO, based on literature findings.

**Table 3:** Multivariable analyses of all three compared groups of MDRO-positive and MDRO-negative patients, by type of pathogen, Charité University Hospital, Germany, June 2019–September 2022 (MRSA: n = 348; VRE: n = 810; MDR-GNB: n = 1,593)

Characteristic		MRSA (n = 348)			VRE (n = 810)			MDR-GNB (n = 1,593)		
		OR	95% CI	p value	OR	95% CI	p value	OR	95% CI	p value
Pet ownership		0.66	0.34–1.28	0.218	0.76	0.52–1.12	0.166	0.82	0.62–1.08	0.160
Sex (male)		1.13	0.71–1.79	0.605	**1.36**	**1.00–1.85**	**0.049**	1.16	0.94–1.44	0.174
Age^a^ in years		1.01	0.99–1.02	0.303	**1.01**	**1.00–1.02**	**0.027**	**1.01**	**1.01–1.02**	**0.001**
Living situation	Family/shared flat	1	Reference		1	Reference		1	Reference	
	Alone	1.15	0.69–1.93	0.588	0.84	0.59–1.18	0.302	0.95	0.74–1.21	0.654
	Nursing home	2.20	0.47–10.24	0.316	2.83	0.92–8.65	0.069	1.51	0.52–4.38	0.444
	Others	NA			3.99	0.35–45.17	0.264	3.42	0.31–38.30	0.319
Professional contact with livestock or pets		1.17	0.41–3.34	0.769	Not included			0.99	0.60–1.63	0.977

CI: confidence interval; DAG: directed acyclic graphs; MDR-GNB: multidrug-resistant Gram-negative bacteria; MRSA: meticillin-resistant *Staphylococcus aureus*; NA: not available due to low number of events; OR: odds ratio; VRE: vancomycin-resistant enterococci.

Analyses were performed based on prospectively modelled DAGs. We analysed pet ownership adjusted for age and sex of the participant as well as potential confounders identified in DAGs, which were living situation and professional contact with livestock or pets for MRSA and MDR-GNB and living situation for VRE. Statistically significant results are highlighted in bold.

^a^ OR represents a difference of 1 year of age.

As pet ownership was associated with decreased risk for MDROs in univariable and multivariable analysis (although not statistically significant), we aimed to assess the potential selection bias for this characteristic. Therefore, we additionally compared pet owning participants (n = 698) and not pet-owning participants (n = 2,192) regarding MDRO colonisation and statistically significant differences in the characteristics of these two groups (n = 2,890; one patient excluded due to inconclusive answer on pet ownership). Pet owners were on average younger (mean age: 56 years in pet owners, 65 years in non-pet owners, p < 0.001), more often female (50.0% males in pet owners, 57.4% males in non-pet owners, p > 0.001) and had fewer comorbidities (mean CCI: 3.0 in pet owners, 4.0 in non-pet owners, p < 0.001). They were less likely to live in nursing homes (0.2% in pet owners, 1.6% in non-pet owners, p < 0.001) and more likely to have professional contact with livestock or pets (14.5% in pet owners, 3.2% in non-pet owners, p < 0.001). Diarrhoea was more prevalent (31.5% in pet owners, 25.3% in non-pet owners, p < 0.001), whereas heart disease occurred less often in pet owners (6.2% in pet owners, 9.6% in non-pet owners, p = 0.008). The complete set of variables of this comparison can be found in Supplementary Table S8.

### Multidrug-resistant organisms transmission between pets and owners

Among the 2,890 study participants with a conclusive answer about pet ownership, 626 (21.7%) owned at least one dog or cat (range: 1–11; mean: 1.5 pets per household). We included 293 dog owners, 266 cat owners and 67 patients living with dogs as well as cats (patients were counted as dog owners as well as cat owners in the following calculations). In total, 154 (17.7%) of MDRO-positive and 472 (23.4%) of MDRO-negative participants were pet owners. Among MDRO carriers, 10.6% were dog owners (93/871) and 9.2% were cat owners (80/871), whereas among MDRO-negative patients, 13.2% were dog owners (267/2,020), 12.5% were cat owners (253/2,020) and one person gave an inconclusive answer about pet ownership. About 4% (n = 115) of all participants owned at least one type of small animal other than dogs or cats; they were 37 with small mammals, 44 with birds, 14 with exotic pets and 30 with fish (multiple counting possible). Most participants who had contact with livestock and/or professional contact with pets had contact with horses (n = 79), followed by poultry (n = 65), sheep or goats (n = 11), cattle (n = 10), pigs (n = 8) and others (n = 15).

**Figure 2:**
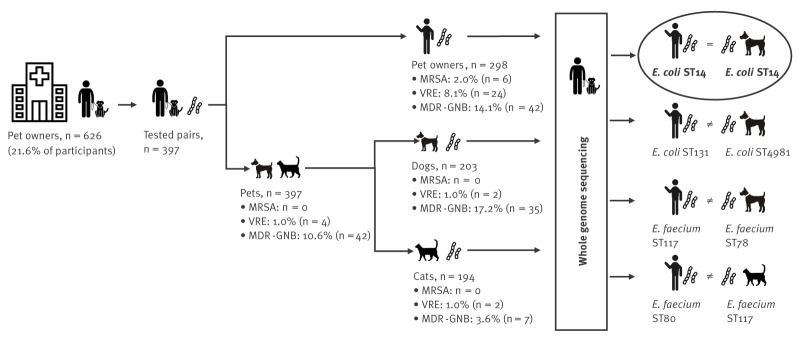
Transmission of multidrug-resistant organisms between pets and owners, Charité University Hospital, Germany, June 2019–September 2022 (n = 397 pairs)

MDR-GNB: multidrug-resistant Gram-negative bacteria; MDRO: multidrug-resistant organism; MRSA: meticillin-resistant *Staphylococcus aureus*; ST: sequence type; VRE: vancomycin-resistant enterococci.

Among the 626 included dog and cat owners, 614 received a screening kit to test their pets at home and 298 of them (48.5%) returned samples from 397 pets, including those of 203 dogs and 194 cats (Figure 2). The median time between sampling of pet owners and pets was 52 days (range: 11–270 days). For 196 of 203 dogs, stool samples and throat swabs were available, for five dogs only stool samples and for two dogs only throat swabs were available. For 175 of 194 cats, stool and swab samples were available, for three cats only stool samples and for 16 cats only throat swab samples were available. In total, 18.2% (37/203) of the sampled dogs and 4.6% (9/194) of the sampled cats were MDRO-positive. Among throat swab samples, we did not identify MRSA. Four stool swab samples tested positive for VRE (two dogs, two cats). All VRE isolates belonged to the species *E. faecium*. The prevalence of VRE was 1.5% in dogs and 1.0% in cats. The 42 MDR-GNB-positive samples were taken from 35 dogs and seven cats. They included 34 ESBL-positive pathogens (30 in dogs, four in cats). The most frequently isolated species among the MDR-GNB isolates was *E. coli* (n = 31), followed by *Enterobacter cloacae* complex (n = 5) and *Citrobacter freundii* (n = 3). *Citrobacter braakii, K. pneumoniae*, and *Proteus penneri* were each detected once. No CR-GNB were isolated from pet samples. We detected fluoroquinolone resistance in 16 of the *E. coli* isolates and in the single *K. pneumoniae* isolate. In total, 17.2% of the sampled dogs and 3.6% of the sampled cats were colonised with MDR-GNB.

Out of 626 pet owners included in the study cohort, we tested 397 owner–pet pairs for MDRO carriage. In total, 72 pet owners and 46 pets tested positive for MDRO colonisation. Phenotypically indistinguishable MDROs were identified in four of 397 owner–pet pairs (1.0%). The bold text highlights the only owner–pet pair sharing *E. coli* ST14 with identical cgMLST types (0.25% of all pairs).

Four pairs of pets and owners were colonised with phenotypically indistinguishable MDROs, comprising two pairs of VR *E. faecium* carriers and two pairs of 3GCR *E. coli* carriers (Figure 2). Sequencing of isolates from all four owner–pet pairs showed the following sequence types (ST) and cgMLST complex types (CT) (Table 4). Therefore, co-carriage of MDROs with identical cgMLST types in pet and owner was observed in 0.25% (1/397) of all sampled owner–pet pairs.

**Table 4:** Owner–pet pairs colonised with phenotypically indistinguishable multidrug-resistant organisms, Charité University Hospital, Germany, June 2019–September 2022 (n = 4)

	Pet		Owner	CT	Allelic distance
Pair 1	Dog	3GCR *E. coli* ST14	3GCR *E. coli* ST14	Not named	≤ 3
Pair 2	Dog	3GCR *E. coli* ST4981	3GCR *E. coli* ST131	Not identical and not named	2,363
Pair 3	Dog	VR *E. faecium* ST78 CT894	VR *E. faecium* ST117 CT 929	Not defined	653
Pair 4	Cat	VR *E. faecium* ST117	VR *E. faecium* ST80	Not identical and not named	140

CT: cgMLST complex type; ST: sequence type.

## Discussion

Infections with MDRO pose an important health risk [2]. To ensure a quick detection and appropriate treatment of MDRO infection, it is crucial to be aware of risk factors for MDRO colonisation. In an urban, high-income setting, neither cat nor dog ownership appeared as a relevant risk factor for MDRO carriage in hospital patients.

In this study, the prevalence of MDR-GNB colonisation in dogs and cats was with 10.6% comparable to the known MDR-GNB prevalence of hospital patients in Germany (9.5–12.7%) [19,32]. Colonisation of the analysed pets with VRE was rare (1% of tested pets), but also comparable to the VRE admission prevalence in hospital patients in Germany (1.4–1.5%) [20,33]. Colonisation with MRSA was not found in the household pets examined, whereas the admission prevalence in German patients is estimated at 1.1–2.0% [34,35]. The prevalence of MDROs in pets also matched published prevalence data for hospital patients [30,36].

Co-carriage of genetically indistinguishable MDROs in pets and owners was extremely rare and found only once in the ca 400 owner–pet-pairs. We identified one additional pair of dog and owner carrying VR *E. faecium*, but did not include it in the analyses since the human swab was performed after the defined timeframe for community acquisition and thus did not meet the strict inclusion criteria. However, this patient and the dog living in the same household were colonised with VR *E. faecium* ST117, CT36 isolates with an allelic distance of ≤ 3 alleles. Only one dog with MRSA colonisation was identified in the complete study cohort, which was also excluded from these analyses, again because the owner of the dog did not meet the inclusion criteria (patient sampled > 72 h after hospital admission).

In the risk factor analysis of the entire sample, we did not identify pet ownership as a statistically significant risk factor for MDRO colonisation in hospital patients. We even found that pet ownership was associated with lower MDRO colonisation rates compared with patients without pets, which may be explained by the younger age and lower comorbidity scores of these patients. Supporting this result, a recent systematic meta-analysis of pet ownership and MDRO colonisation found no relevant association between pet ownership and MDR-GNB colonisation. The meta-analysis did find an association between dog ownership and MRSA colonisation, but this was highly dependent on results of one study in persons living on pig farms and therefore being at higher risk of MRSA carriage than dog owners without contact with livestock [13].

Contact with livestock was rare in our cohort as we recruited patients in an urban setting. Therefore, in cohorts from rural areas with more contact with livestock, the results might be different. Moreover, we rarely found MRSA in our hospital patients and not at all in the household pets analysed. This agrees with the results of a previous study, in which only three MRSA were found among 803 dogs and 117 cats sampled at admittance to a small animal clinic during a period of 17 months [10]. A more recent study identified *S. aureus* in 3.2 % of 175,171 canine and feline samples from German veterinary practices. Among them, 17.8% showed meticillin resistance [37]. Thus, our study was probably not sufficiently powered to detect risk factors for MRSA colonisation. However, the MRSA prevalence in northern Europe and Germany has declined continuously during the past 20 years, and our results support this observation [38,39]. Codependent on different risk factors, such as long-term catheter use, MRSA prevalence is currently estimated at ca 2.0% at hospital admission in general [34,40]. Our study confirmed established risk factors for MRSA colonisation in the univariable analysis. The meta-analysis by Hackmann et al. found no association concerning MDR-GNB and insufficient data on VRE colonisation to analyse its association to pets [13]. Our results for VRE and MDR-GNB support this observation. Regarding MDR-GNB colonisation, we found an association with travel to Asia. This has been shown in various other studies and seems to be an expression of a high colonisation pressure deriving from high MDR-GNB prevalence in the general population in that region [25,41,42]. Further, we found an association between cancerous diseases and VRE colonisation. Literature also shows that patients with cancer are known to have a higher risk for VRE colonisation [24]. This is observed mainly for leukaemia patients and is most probably a result of repeated antimicrobial therapy and multiple hospital stays in the patients’ recent past. It has been suggested that this observation derives from the fact that vancomycin is often included in the empirical therapy of neutropenic fever [24].

Our study does not allow an estimation of the direction of transmission between pets and owners. Transmission of MDRO can occur from owner to pet and vice versa. In addition, transmission can go back and forth between pets and owners living in the same household. In this study, the colonisation status was based on a single sampling occasion, therefore, no estimation of the beginning of the colonisation period for pet or owner was possible. Multiple follow-up screening of pets and owners might have increased transmission detection. Dazio et al. performed a longitudinal analysis of MDRO colonisation in pets and owners but did not observe owner–pet co-carriage [43].

Our study was conducted in a specific setting. We included hospital patients in an urban environment of a high-income country. As we included hospital patients, human study subjects were older and more often male than the general population. Results might therefore be different in other settings, notably low- or middle-income countries with different sociocultural backgrounds. Moreover, the frequency of pet ownership in our study group was lower than expected based on the frequency in the general population. We assume that pet ownership is less common in the hospital patient cohort because of higher age, a higher frequency of chronic and severe diseases as well as living in care facilities. In addition, pet ownership in urban areas might be less frequent than in rural communities.

Another potential limitation of this study is the possible under-detection of MDRO carriage in pets due to problems in taking samples and further processing of the samples. Oral and faecal swabs taken by the owner and sent to a laboratory via postal shipping is a feasible method to examine colonisation with Gram-positive and Gram-negative bacterial pathogens in dogs and cats [44]. However, participants reported that taking swab samples from the throat of their pets was challenging, especially in cats. Furthermore, taking stool samples of cats living mostly outside posed a problem to cat owners. In addition, a long shipping time of samples, as we experienced in this study, could have led to an overgrowth of bacteria and therefore to an under-reporting of resistant bacteria. We did not include further enrichment steps that could have increased the sensitivity of the tests.

This study was performed between 2019 and 2022 and was therefore affected by the challenges and restrictions of the COVID-19 pandemic. Recruitment of patients had to be paused during lockdowns and diseases in the patient cohort were more severe because elective surgeries were postponed. Infection prevention measures were intensified in the health system as well as the private sector. Physical contact and travelling were reduced, hospital visits were limited [45]. These changes may have affected the results of this study, it was, for example, not possible to reach the planned number of participants for this study in the given time. 

Furthermore, we decided on very strict inclusion criteria for our analyses and accepted the additional reduction in the number of included participants to reach a high quality of data. However, this is still a large study and the data show that we were able to identify the established risk factors for the different MDRO species. Therefore, we think the study design and power was adequate to assess the risk of pet ownership for MDRO colonisation in hospital patients. 

## Conclusion

We found overall similar colonisation rates for MDR-GNB and VRE in pets as known for hospital patients in a high-income urban setting. In the risk factor analysis, we did not find pet ownership to be a statistically significant risk factor for MDRO colonisation in hospital patients. We demonstrated that MDRO transmission between hospital patients and their pets is possible, but extremely rare. The evidence from our study will help to further improve the identification of patients at high risk for MDRO colonisation and the empirical antimicrobial treatment strategy in hospital patients. It does not indicate that pet ownership should be included as a risk factor for MDRO colonisation. Still, transmission between owners and pets should be kept in mind especially for patients with recurrent infections or persistent colonisation. Our study further promotes the application of the One Health approach, especially in the field of antimicrobial resistance.

## Supplementary Data

375000 Supplement

## Supplementary Data

513000 Supplement1
