# Supplementary material for: The transmission risk of multidrug-resistant organisms between hospital patients and their pets – a case−control study, Germany, 2019 to 2022
Source: Euro Surveill. 2024 Sep 26;29(39):2300714. doi: 10.2807/1560-7917.ES.2024.29.39.2300714 (PMC11484343; doi:10.2807/1560-7917.ES.2024.29.39.2300714)
Supplement: Supplement [file 23-00714_HACKMANN_Supplement.pdf]

Supplement

Supplementary tables and figures for univariable analyses, resistance pattern of isolates and DAGs created for multivariable analysis

This supplementary material is hosted by Eurosurveillance as supporting information alongside the article "The transmission risk of multidrug-resistant organisms (MDRO) between hospital patients and their pets – a case control study", on behalf of the authors, who remain responsible for the accuracy and appropriateness of the content. The same standards for ethics, copyright, attributions and permissions as for the article apply. Supplements are not edited by Eurosurveillance and the journal is not responsible for the maintenance of any links or email addresses provided therein.

Table S1 - Distribution of collected data characteristics in the study cohort. BMI = Body Mass Index, CCI = Charlson Comorbidity Index, N = available data sets for each characteristic, *Median (Interquartile range (IQR)).

Characteristic	N	Overall, N = 2,891	MDRO-Positive, N = 871	MDRO-Negative, N = 2,020
Sex	2,891			
Male		1,608 (56%)	507 (58%)	1,101 (55%)
Female		1,282 (44%)	364 (42%)	918 (45%)
Non-binary		1 (<0.1%)	0 (0%)	1 (<0.1%)
Age*	2,891	63 (51, 73)	65 (53, 74)	61 (50, 72)
BMI*	2,891	24.8 (22.0, 28.7)	24.5 (21.6, 28.3)	25.1 (22.2, 29.0)
CCI*	2,876	4 (2, 7)	4 (2, 7)	4 (2, 6)
Diet	2,888			
Mixed diet		2,747 (95%)	836 (96%)	1,911 (95%)
Vegetarian		99 (3.4%)	18 (2.1%)	81 (4.0%)
Vegan		12 (0.4%)	5 (0.6%)	7 (0.3%)
Others		30 (1.0%)	9 (1.0%)	21 (1.0%)
Living situation	2,888			
Family/shared flat		2,041 (71%)	609 (70%)	1,432 (71%)
Alone		805 (28%)	239 (28%)	566 (28%)
Nursing Home		37 (1.3%)	18 (2.1%)	19 (0.9%)
Others		5 (0.2%)	2 (0.2%)	3 (0.1%)
Prior hospitalization	2,878	1,764 (61%)	649 (75%)	1,115 (55%)
Prior antibiotic use	2,815	1,187 (41%)	543 (62%)	644 (32%)
Travel within Europe	2,890	403 (14%)	111 (13%)	292 (14%)
Travel to Asia	2,889	103 (3.6%)	46 (5.3%)	57 (2.8%)
Travel to North America	2,889	24 (0.8%)	11 (1.3%)	13 (0.6%)
Travel to South America	2,889	24 (0.8%)	11 (1.3%)	13 (0.6%)
Travel to Africa	2,889	24 (0.8%)	10 (1.1%)	14 (0.7%)
Travel to Australia/New Zealand	2,889	2 (<0.1%)	0 (0%)	2 (<0.1%)
Urinary tract catheter	2,888	291 (10%)	117 (13%)	174 (8.6%)
Central venous catheter	2,888	612 (21%)	257 (30%)	355 (18%)
Prior MDR-GNB colonization	2,434	148 (5.1%)	99 (11%)	49 (2.4%)
Prior MRSA colonization	2,433	90 (3.1%)	61 (7.1%)	29 (1.4%)
Prior VRE colonization	2,432	69 (2.4%)	42 (4.9%)	27 (1.3%)
Diarrhea	2,725	771 (27%)	280 (32%)	491 (24%)
Pet ownership†	2,890	626 (22%)	154 (18%)	472 (23%)
Dog ownership	2,890	360 (12%)	93 (11%)	267 (13%)
Cat ownership	2,890	333 (12%)	80 (9.2%)	253 (13%)
Ownership of other pets	2,890	115 (4.0%)	36 (4.1%)	79 (3.9%)
Regular contact to pets of others	2,888	159 (5.5%)	32 (3.7%)	127 (6.3%)
Number of dogs	2,891			
0		2,532 (88%)	779 (89%)	1,753 (87%)
1		309 (11%)	83 (9.5%)	226 (11%)
2		38 (1.3%)	7 (0.8%)	31 (1.5%)
3 or more		12 (0.4%)	2 (0.2%)	10 (0.5%)
Number of cats	2,891			
0		2,561 (89%)	793 (91%)	1,768 (88%)

Characteristic	N	Overall, N = 2,891	MDRO-Positive, N = 871	MDRO-Negative, N = 2,020
1		222 (7.7%)	54 (6.2%)	168 (8.3%)
2		80 (2.8%)	16 (1.8%)	64 (3.2%)
3 or more		28 (1.0%)	8 (0.9%)	20 (1.0%)
Professional contact to livestock or pets	2,882	163 (5.7%)	43 (5.0%)	120 (6.0%)
Heart disease	2,876	257 (8.9%)	82 (9.5%)	175 (8.7%)
Cerebrovascular disease	2,876	195 (6.8%)	62 (7.2%)	133 (6.6%)
Neurologic disease	2,876	51 (1.8%)	23 (2.7%)	28 (1.4%)
Lung disease	2,876	251 (8.7%)	65 (7.5%)	186 (9.2%)
Rheumatic disease	2,876	102 (3.5%)	28 (3.2%)	74 (3.7%)
Gastrointestinal disease	2,876	24 (0.8%)	11 (1.3%)	13 (0.6%)
Liver disease	2,876	163 (5.7%)	54 (6.2%)	109 (5.4%)
Diabetes	2,876	450 (16%)	144 (17%)	306 (15%)
Renal disease	2,876	672 (23%)	263 (30%)	409 (20%)
Cancer/immunological disease	2,876	1,074 (37%)	371 (43%)	703 (35%)

Table S2 Resistance pattern of isolates from participating hospital patients, n = 985. ESBL = Extended Spectrum Beta-Lactamase.

	**Total**	**Methicillin resistant**	**Vancomycin resistant**	**3rd Generation Cephalosporine resistant**	**ESBL positive**	**Fluoroquinolone resistant**	**Carbapenem resistant**
**Staphylococci**							
Staphylococcus aureus	118	118	-	-	-	-	-
**Enterococci**							
Enterococcus faecium	273	-	273	-	-	-	-
Enterococcus faecalis	4	-	4	-	-	-	-
**Gram-negative bacteria**							
Escherichia coli	411	-	-	411	378	310	4
Klebsiella pneumoniae	102	-	-	102	85	78	6
Enterobacter cloacae complex	26	-	-	26	11	2	2
Citrobacter freundii	18	-	-	18	7	6	1
Pseudomonas aeruginosa	9	-	-	9	2	9	3
Acinetobacter baumanni	2	-	-	2	0	2	2
Enterobacter aerogenes	2	-	-	2	0	0	0
Klebsiella oxytoca	8	-	-	8	6	2	0
Morganella morganii	2	-	-	2	0	2	0
Proteus mirabilis	2	-	-	2	1	2	0
Raoultella planticola	1	-	-	1	0	1	0
Serratia marcescens	1	-	-	1	0	1	0
Other *Citrobacter* species	6	-	-	6	1	1	0
**Sum**	**985**	**118**	**277**	**590**	**491**	**416**	**18**

Table S3 – Complete univariable analysis of matched group of MRSA-positives and MRSA-negatives. N = available data sets included for each characteristic, OR = Odds Ratio, 95% CI = 95% Confidence interval. BMI = Body Mass Index, CCI = Charlson Comorbidity Index, *Median (Interquartile range (IQR)), NA = not available. † Pets = dogs and/or cats, NA = not available due to low number of events.

Characteristic	N	MRSA-Positive, N = 117	MRSA-Negative, N = 234	OR	95% CI	p-value
Sex (male)	351	73 (62%)	139 (59%)	1.124	0.723, 1.745	0.604
Age*	351	64 (50, 76)	60 (46, 73)	1.010	0.998, 1.023	0.100
BMI*	351	24.0 (22.0, 27.5)	24.7 (21.7, 28.6)	0.996	0.958, 1.035	0.845
CCI*	350	5 (2, 8)	4 (1, 7)	1.044	0.982, 1.110	0.167
Diet	351					
Mixed diet		114 (97%)	217 (93%)	1	-	-
Vegetarian		0 (0%)	11 (4.7%)	NA	NA	NA
Vegan		1 (0.9%)	1 (0.4%)	2.000	0.125, 31.975	0.624
Others		2 (1.7%)	5 (2.1%)	0.800	0.155, 4.123	0.790
Living situation	351					
Family/shared flat		74 (63%)	162 (69%)	1	-	-
Alone		39 (33%)	68 (29%)	1.229	0.752, 2.008	0.410
Nursing Home		4 (3.4%)	3 (1.3%)	2.751	0.614, 12.322	0.186
Others		0 (0%)	1 (0.4%)	NA	NA	NA
Prior hospitalization	349	79 (68%)	128 (55%)	1.871	1.131, 3.096	0.015
Prior antibiotic use	336	71 (61%)	85 (36%)	2.596	1.619, 4.162	<0.001
Travel within Europe	351	10 (8.5%)	30 (13%)	0.647	0.309, 1.355	0.248
Travel to Asia	351	6 (5.1%)	8 (3.4%)	1.618	0.505, 5.185	0.418
Travel to North America	351	3 (2.6%)	2 (0.9%)	3.000	0.501, 17.954	0.229
Travel to South America	351	3 (2.6%)	2 (0.9%)	3.000	0.501, 17.954	0.229
Travel to Africa	351	2 (1.7%)	2 (0.9%)	2.000	0.282, 14.198	0.488
Travel to Australia/New Zealand	351	0 (0.0%)	2 (0.9%)	NA	NA	NA
Urinary tract catheter	351	9 (7.7%)	16 (6.8%)	1.144	0.477, 2.740	0.763
Central venous catheter	351	29 (25%)	34 (15%)	2.289	1.214, 4.319	0.011
Prior MDR-GNB colonization	304	3 (2.6%)	5 (2.1%)	1.500	0.336, 6.702	0.595
Prior MRSA colonization	305	48 (41%)	5 (2.1%)	86.098	11.870, 624.510	<0.001
Prior VRE colonization	305	1 (0.9%)	5 (2.1%)	0.549	0.056, 5.392	0.607
Diarrhea	348	31 (26%)	54 (23%)	1.179	0.702, 1.981	0.533
Pet ownership†	351	18 (15.4%)	55 (23.5%)	0.58	0.32, 1.06	0.075
Dog ownership	351	12 (10%)	31 (13%)	0.745	0.365, 1.518	0.417
Cat ownership	351	7 (6.0%)	30 (13%)	0.432	0.183, 1.017	0.055
Ownership of other pets	351	4 (3.4%)	12 (5.1%)	0.632	0.191, 2.092	0.453
Regular contact to pets of others	351	3 (2.6%)	14 (6.0%)	0.429	0.123, 1.491	0.183
Number of dogs	351					
0		105 (90%)	203 (87%)	1	-	-
1		11 (9.4%)	27 (12%)	0.761	0.364, 1.594	0.470
2		0 (0%)	4 (1.7%)	NA	NA	NA
3 or more		1 (0.9%)	0 (0%)	NA	NA	NA
Number of cats	351					
0		110 (94%)	204 (87%)	1		
1		5 (4.3%)	21 (9.0%)	0.448	0.168, 1.194	0.109
2		1 (0.9%)	5 (2.1%)	0.356	0.041, 3.071	0.348
3 or more		1 (0.9%)	4 (1.7%)	0.436	0.048, 3.936	0.460
Professional contact to livestock or pets	350	6 (5.2%)	15 (6.4%)	0.791	0.301, 2.084	0.636
Heart disease	349	16 (14%)	19 (8.2%)	1.979	0.901, 4.346	0.089
Cerebrovascular disease	349	11 (9.5%)	17 (7.3%)	1.391	0.588, 3.291	0.453
Neurologic disease	349	8 (6.9%)	4 (1.7%)	4.000	1.205, 13.283	0.024
Lung disease	349	9 (7.8%)	26 (11%)	0.655	0.292, 1.470	0.305
Rheumatic disease	349	5 (4.3%)	9 (3.9%)	1.120	0.360, 3.490	0.845
Gastrointestinal disease	349	1 (0.9%)	0 (0%)	NA	NA	NA
Liver disease	349	9 (7.8%)	9 (3.9%)	2.099	0.802, 5.490	0.131
Diabetes	349	20 (17%)	29 (12%)	1.412	0.769, 2.590	0.266

Characteristic	N	MRSA-Positive, N = 117	MRSA-Negative, N = 234	OR	95% CI	p-value
Renal disease	349	37 (32%)	61 (26%)	1.334	0.799, 2.230	0.271
Cancer/immunological disease	349	49 (42%)	86 (37%)	1.267	0.780, 2.060	0.339

Table S4 - Complete univariable analysis of matched group of VRE-positives and VRE-negatives. N = number of data sets included for each characteristic, OR = Odds Ratio, 95% CI = 95% Confidence interval. BMI = Body Mass Index, CCI = Charlson Comorbidity Index, N = available data sets for each characteristic, *Median (Interquartile range (IQR)). † Pets = dogs and/or cats, NA = not available due to low number of events.

Characteristic	N	VRE-Positive, N = 275	VRE-Negative, N = 550	OR	95% CI	p-value
Sex (male)	825	172 (63%)	300 (55%)	1.392	1.034, 1.875	0.029
Age*	825	66 (55, 73)	62 (53, 71)	1.014	1.004, 1.024	0.007
BMI*	825	24.1 (21.1, 27.8)	25.1 (21.8, 28.4)	0.971	0.945, 0.998	0.036
CCI*	823	5 (3, 7)	4 (2, 7)	1.058	1.016, 1.102	0.006
Diet	822					
Mixed diet		258 (95%)	510 (93%)	1	-	-
Vegetarian		6 (2.2%)	24 (4.4%)	0.499	0.198, 1.257	0.140
Vegan		2 (0.7%)	3 (0.5%)	1.333	0.223, 7.980	0.753
Others		6 (2.2%)	13 (2.4%)	0.904	0.335, 2.441	0.842
Living situation	823					
Family/shared flat		193 (71%)	386 (70%)	1	-	-
Alone		69 (25%)	158 (29%)	0.846	0.607, 1.181	0.326
Nursing Home		9 (3.3%)	5 (0.9%)	3.519	1.178, 10.512	0.024
Others		2 (0.7%)	1 (0.2%)	3.896	0.353, 4.3001	0.267
Prior hospitalization	820	241 (88%)	368 (67%)	3.921	2.533, 6.069	<0.001
Prior antibiotic use	762	188 (68%)	206 (38%)	5.111	3.460, 7.550	<0.001
Travel within Europe	825	27 (9.8%)	70 (13%)	0.748	0.468, 1.195	0.224
Travel to Asia	824	6 (2.2%)	18 (3.3%)	0.644	0.247, 1.681	0.369
Travel to North America	824	3 (1.1%)	5 (0.9%)	1.200	0.287, 5.021	0.803
Travel to South America	824	3 (1.1%)	5 (0.9%)	1.200	0.287, 5.021	0.803
Travel to Africa	824	1 (0.4%)	4 (0.7%)	0.500	0.056, 4.473	0.535
Travel to Australia/New Zealand	824	0 (0.0%)	0 (0.0%)	NA	NA	NA
Urinary tract catheter	824	36 (13%)	42 (7.7%)	2.217	1.275, 3.853	0.005
Central venous catheter	824	99 (36%)	152 (28%)	1.619	1.141, 2.297	0.007
Prior MDR-GNB colonization	654	11 (4.0%)	8 (1.5%)	3.922	1.353, 11.365	0.012
Prior MRSA colonization	654	10 (3.7%)	4 (0.7%)	4.893	1.310, 18.280	0.018
Prior VRE colonization	652	33 (12%)	12 (2.2%)	10.164	3.924, 26.329	<0.001
Diarrhea	818	109 (40%)	141 (26%)	2.064	1.487, 2.866	<0.001
Pet ownership†	825	49 (17.8%)	131 (23.8%)	0.69	0.48, 1.00	0.050
Dog ownership	825	27 (9.8%)	80 (15%)	0.632	0.396, 1.011	0.056
Cat ownership	825	30 (11%)	67 (12%)	0.883	0.559, 1.394	0.594
Ownership of other pets	825	14 (5.1%)	27 (4.9%)	1.038	0.540, 1.996	0.911
Regular contact to pets of others	823	11 (4.0%)	29 (5.3%)	0.748	0.368, 1.522	0.424
Number of dogs	825					
0		249 (91%)	470 (85%)	1	-	-
1		25 (9.1%)	70 (13%)	0.659	0.406, 1.071	0.092
2		0 (0%)	8 (1.5%)	NA	NA	NA
3 or more		1 (0.4%)	2 (0.4%)	0.938	0.085, 10.381	0.959
Number of cats	825					
0		246 (89%)	484 (88%)	1	-	-
1		22 (8.0%)	43 (7.8%)	1.003	0.592, 1.702	0.990
2		6 (2.2%)	13 (2.4%)	0.923	0.350, 2.436	0.872
3 or more		1 (0.4%)	10 (1.8%)	0.200	0.026, 1.564	0.125
Professional contact to livestock or pets	822	15 (5.5%)	37 (6.7%)	0.794	0.423, 1.488	0.471

Characteristic	N	VRE-Positive, N = 275	VRE-Negative, N = 550	OR	95% CI	p-value
Heart disease	823	29 (11%)	38 (6.9%)	1.627	0.965, 2.745	0.068
Cerebrovascular disease	823	22 (8.0%)	27 (4.9%)	1.711	0.945, 3.099	0.076
Neurologic disease	823	6 (2.2%)	6 (1.1%)	2.156	0.647, 7.190	0.211
Lung disease	823	25 (9.1%)	50 (9.1%)	1.000	0.590, 1.696	>0.900
Rheumatic disease	823	9 (3.3%)	14 (2.6%)	1.259	0.533, 2.976	0.600
Gastrointestinal disease	823	4 (1.5%)	5 (0.9%)	1.600	0.430, 5.958	0.484
Liver disease	823	22 (8.0%)	28 (5.1%)	1.642	0.912, 2.955	0.098
Diabetes	823	43 (16%)	77 (14%)	1.140	0.759, 1.711	0.529
Renal disease	823	82 (30%)	110 (20%)	1.714	1.222, 2.404	0.002
Cancer/immunological disease	823	148 (54%)	293 (44%)	1.662	1.203, 2.295	0.002

Table S5 - Complete univariable analysis of matched group of MDR-GNB-positives and MDR-GNB-negatives. N = number of data sets included for each characteristic, OR = Odds Ratio, 95% CI = 95% Confidence interval. BMI = Body Mass Index, CCI = Charlson Comorbidity Index, N = available data sets for each characteristic, *Median (Interquartile range (IQR)). † Pets = dogs and/or cats, NA = not available due to low number of events.

Characteristic	N	MDR-GNB-Positive, N = 534	MDR-GNB-Negative, N = 1,068	OR	95% CI	p-value
Sex (male)	1602	301 (56%)	560 (52%)	1.175	0.952, 1.451	0.133
Age*	1602	65 (53, 74)	61 (50, 71)	1.014	1.007, 1.021	<0.001
BMI*	1602	24.5 (21.6, 28.7)	25.1 (22.0, 28.7)	0.991	0.973, 1.010	0.367
CCI*	1592	4 (2, 7)	4 (2, 6)	1.033	1.003, 1.065	0.032
Diet	1601					
Mixed diet		515 (97%)	1,016 (95%)	1	-	-
Vegetarian		12 (2.3%)	41 (3.8%)	0.574	0.300, 1.099	0.094
Vegan		3 (0.6%)	1 (<0.1%)	6.000	0.624, 57.681	0.121
Others		3 (0.6%)	10 (0.9%)	0.577	0.159, 2.100	0.404
Living situation	1601					
Family/shared flat		382 (72%)	768 (72%)	1	-	-
Alone		142 (27%)	292 (27%)	0.979	0.776, 1.236	0.859
Nursing Home		7 (1.3%)	7 (0.7%)	1.988	0.696, 5.679	0.200
Others		2 (0.4%)	1 (<0.1%)	3.986	0.361, 43.972	0.259
Prior hospitalization	1596	372 (70%)	628 (59%)	1.703	1.342, 2.161	<0.001
Prior antibiotic use	1504	318 (60%)	348 (33%)	4.317	3.305, 5.639	<0.001
Travel within Europe	1601	79 (15%)	171 (16%)	0.910	0.680, 1.217	0.525
Travel to Asia	1601	34 (6.4%)	38 (3.6%)	1.806	1.132, 2.881	0.013
Travel to North America	1601	7 (1.3%)	9 (0.8%)	1.556	0.579, 4.177	0.381
Travel to South America	1601	7 (1.3%)	9 (0.8%)	1.556	0.579, 4.177	0.381
Travel to Africa	1601	7 (1.3%)	8 (0.7%)	1.832	0.634, 5.292	0.264
Travel to Australia/New Zealand	1601	0 (0.0%)	0 (0.0%)	NA	NA	NA
Urinary tract catheter	1600	80 (15%)	70 (6.6%)	2.849	1.963, 4.133	<0.001
Central venous catheter	1600	148 (28%)	222 (21%)	1.651	1.248, 2.185	<0.001
Prior MDR-GNB colonization	1334	91 (17%)	32 (3.0%)	10.467	5.931, 18.473	<0.001
Prior MRSA colonization	1332	8 (1.5%)	13 (1.2%)	1.761	0.655, 4.733	0.262
Prior VRE colonization	1332	10 (1.9%)	18 (1.7%)	1.207	0.519, 2.806	0.662
Diarrhea	1594	169 (32%)	302 (28%)	1.197	0.947, 1.513	0.133
Pet ownership†	1602	99 (18.5%)	248 (23.2%)	0.75	0.57, 0.97	0.030
Dog ownership	1602	61 (11%)	137 (13%)	0.875	0.634, 1.209	0.419
Cat ownership	1602	48 (9.0%)	134 (13%)	0.684	0.481, 0.972	0.034
Ownership of other pets	1602	23 (4.3%)	43 (4.0%)	1.077	0.633, 1.833	0.784
Regular contact to pets of others	1601	21 (3.9%)	77 (7.2%)	0.536	0.329, 0.874	0.012

Characteristic	N	MDR-GNB-Positive, N = 534	MDR-GNB-Negative, N = 1,068	OR	95% CI	p-value
Number of dogs	1602					
0		473 (89%)	931 (87%)	1	-	-
1		54 (10%)	111 (10%)	0.957	0.680, 1.345	0.799
2		7 (1.3%)	21 (2.0%)	0.652	0.273, 1.558	0.335
3 or more		0 (0%)	5 (0.5%)	NA	NA	NA
Number of cats	1602					
0		487 (91%)	934 (87%)	1	-	-
1		30 (5.6%)	102 (9.6%)	0.566	0.370, 0.866	0.009
2		11 (2.1%)	27 (2.5%)	0.773	0.379, 1.573	0.477
3 or more		6 (1.1%)	5 (0.5%)	2.185	0.663, 7.201	0.199
Professional contact to livestock or pets	1599	26 (4.9%)	59 (5.5%)	0.870	0.537, 1.411	0.573
Heart disease	1591	46 (8.7%)	88 (8.3%)	1.051	0.720, 1.536	0.795
Cerebrovascular disease	1591	40 (7.6%)	57 (5.4%)	1.444	0.947, 2.203	0.088
Neurologic disease	1591	11 (2.1%)	14 (1.3%)	1.528	0.693, 3.372	0.294
Lung disease	1591	31 (5.9%)	86 (8.1%)	0.708	0.463, 1.083	0.112
Rheumatic disease	1591	16 (3.0%)	29 (2.7%)	1.127	0.575, 2.208	0.727
Gastrointestinal disease	1591	6 (1.1%)	10 (0.9%)	1.200	0.436, 3.302	0.724
Liver disease	1591	27 (5.1%)	61 (5.7%)	0.871	0.546, 1.388	0.561
Diabetes	1591	88 (17%)	166 (16%)	1.065	0.802, 1.413	0.663
Renal disease	1591	163 (31%)	222 (21%)	1.683	1.328, 2.134	<0.001
Cancer/immunological disease	1591	207 (39%)	399 (38%)	1.100	0.865, 1.399	0.436

Figure S6 Directed acyclic graphs (DAGs) of minimal sufficient adjustment sets constructed for the analysis of the exposure (E) pet ownership on the outcomes (O) MRSA colonization (A), VRE colonization (B) and MDR-GNB colonization (C) based on literature findings (19-31). Authors illustration based on output created in DAGitty software (17).

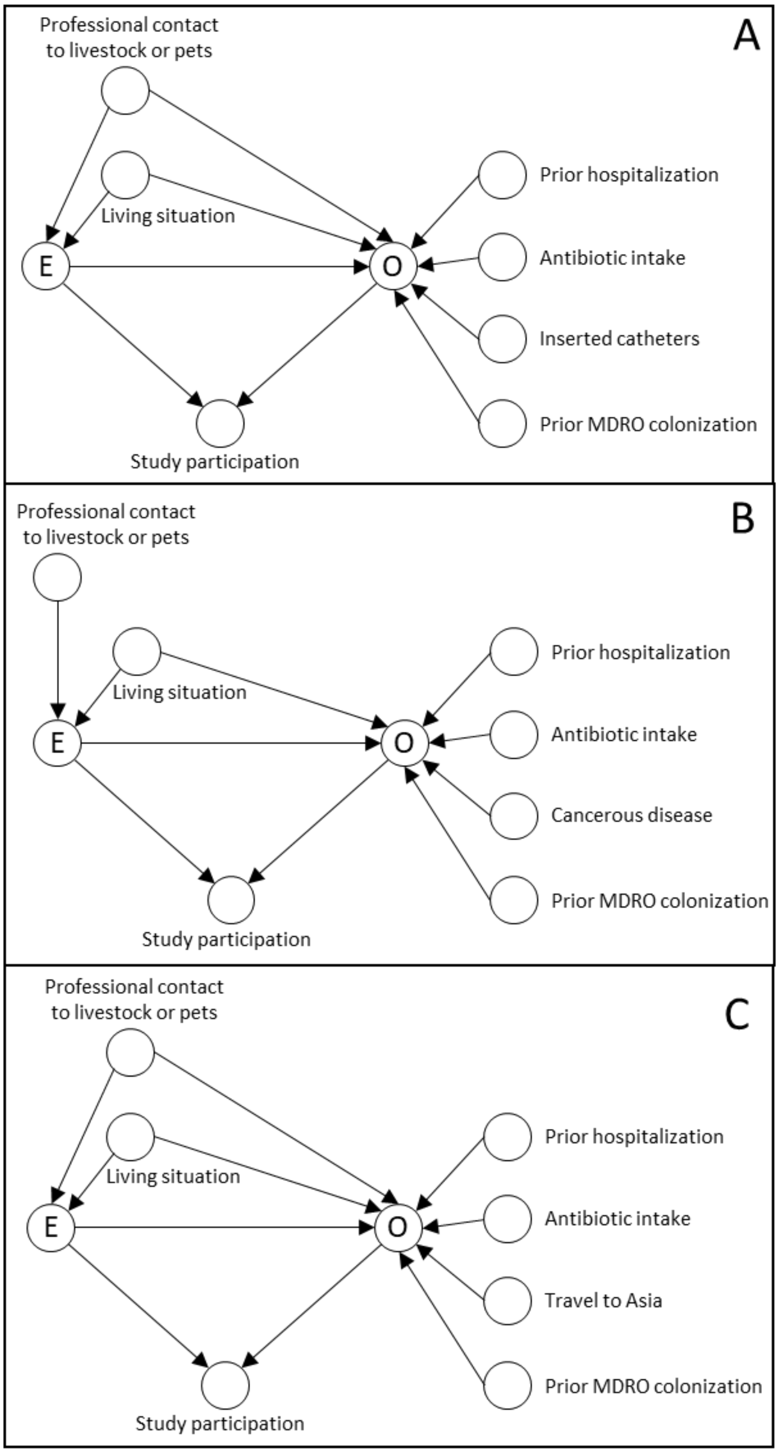

Table S7 - Multivariable analyses of all three compared groups, based on type pf pathogen. Analyses were performed based on prospectively modelled DAGs. We analyzed pet ownership adjusted for age and sex of the participant as well as potential confounders identified in DAGs, which were living situation and professional contact to livestock or pets for MRSA and MDR-GNB, as well as living situation for VRE. In the sensitivity analyses at hand, the most relevant risk factors for each type of MDRO are included in the analyses. They are prior hospitalization and prior antibiotic use for MRSA, VRE and MDR-GNB, travel to Asia for MDR-GNB, cancerous diseases for VRE and inserted catheters for MRSA. Prior colonization with MDROs was not included in these analyses, since we identified a bias in patient recruitment for these variables. We specifically approached patients with a known history of MDRO carriage, therefore we cannot include it as an independent risk factor. *Family/shared flat = reference, OR = Odds Ratio, 95% CI = 95% Confidence interval, NA = not available due to low number of events. Statistically significant results are highlighted (bold). Sensitivity analyses without matching and matching without the multiple use of controls lead to similar results. We do not expect multiple use of controls in matching to have influenced the results.

	MRSA			VRE			MDR-GNB		
**Characteristic**	**OR**	**95% CI**	**p-value**	**OR**	**95% CI**	**p-value**	**OR**	**95% CI**	**p-value**
Pet ownership	0.853	0.415, 1.756	0.667	0.891	0.569, 1.394	0.612	**0.685**	**0.498, 0.943**	**0.020**
Age (median)	1.004	0.989, 1.019	0.607	1.009	0.977, 1.021	0.146	**1.012**	**1.004, 1.020**	**0.005**
Sex (male)	1.107	0.674, 1.817	0.688	1.251	0.863, 1.813	0.237	1.257	0.981, 1.612	0.070
Living situation									
Family/shared flat*	1	-	-	1	-	-	1	-	-
Alone	1.321	0.735, 2.375	0.352	0.928	0.605, 1.423	0.731	0.976	0.738, 1.289	0.863
Nursing home	2.118	0.401, 11.177	0.376	1.321	0.346, 5.044	0.648	2.325	0.580, 9.326	0.234
Others	NA	NA	NA	0.919	0.045, 18.725	0.956	1.787	0.078, 40.808	0.716
Prior hospitalization	1.357	0.764, 2.411	0.298	**2.708**	**1.668, 4.397**	**<0.001**	1.254	0.940, 1.674	0.124
Prior antibiotic use	**2.219**	**1.342, 3.667**	**0.002**	**4.109**	**2.727, 6.192**	**<0.001**	**4.414**	**3.311, 5.886**	**<0.001**
Travel to Asia	-	-	-	-	-	-	**2.403**	**1.408, 4.101**	**0.001**
Professional contact to livestock or pets	0.954	0.295, 3.090	0.938	-	-	-	1.129	0.641, 1.990	0.674
Urinary tract catheter	0.940	0.354, 2.496	0.901	-	-	-	-	-	-
Central venous catheter	1.763	0.852, 3.648	0.126	-	-	-	-	-	-
Cancer	-	-	-	**1.659**	**1.109, 2.482**	**0.014**	-	-	-

Table S8– Complete comparison of pet owners and non-pet owners in this study group, based on p-value (Pearson's Chi-squared test, Fisher's exact test or Wilcoxon rank sum test). N = number of data sets included for each characteristic, *Median (Interquartile range (IQR)), BMI = Body Mass Index, CCI = Charlson Comorbidity Index.

Characteristic	N	Pet ownership, N = 626	Non-pet ownership, N = 2,264	p-value
MDRO colonization	2,890	154 (25%)	717 (32%)	0.001
MRSA colonization	2,890	18 (2.9%)	100 (4.4%)	0.085
VRE colonization	2,890	48 (7.7%)	227 (10%)	0.075
MDR-GNB colonization	2,890	99 (16%)	444 (20%)	0.031
Sex (male)	2,890	313 (50%)	1,294 (57%)	0.001
Age*	2,890	56 (46, 65)	65 (53, 74)	<0.001
BMI*	2,890	25.5 (22.1, 29.1)	24.8 (21.9, 28.7)	0.090
CCI*	2,875	3.0 (1.0, 6.0)	4.0 (2.0, 7.0)	0.001
Diet	2,887			0.332
Mixed diet		590 (94%)	2,156 (95%)	
Vegetarian		27 (4.3%)	72 (3.2%)	
Vegan		4 (0.6%)	8 (0.4%)	
Others		5 (0.8%)	25 (1.1%)	
Living situation	2,887			<0.001
Family/shared flat		522 (83%)	1,519 (67%)	
Alone		103 (16%)	701 (31%)	
Nursing Home		1 (0.2%)	36 (1.6%)	
Others		0 (0%)	5 (0.2%)	
Prior hospitalization	2,886	383 (61%)	1,380 (61%)	0.870
Prior antibiotic use	2,889	271 (43%)	915 (40%)	0.390
Travel within Europe	2,889	93 (15%)	310 (14%)	0.459
Travel to Asia	2,888	18 (2.9%)	84 (3.7%)	0.315
Travel to North America	2,888	3 (0.5%)	21 (0.9%)	0.273
Travel to South America	2,888	3 (0.5%)	21 (0.9%)	0.273
Travel to Africa	2,888	4 (0.6%)	20 (0.9%)	0.550
Travel to Australia/New Zealand	2,888	0 (0%)	2 (<0.1%)	>0.9
Urinary tract catheter	2,887	57 (9.1%)	234 (10%)	0.360
Central venous catheter	2,887	123 (20%)	489 (22%)	0.284
Prior MDR-GNB colonization	2,433	28 (4.5%)	120 (5.3%)	0.458
Prior MRSA colonization	2,432	14 (2.2%)	756(3.4%)	0.274
Prior VRE colonization	2,431	17 (2.7%)	52 (2.3%)	0.635
Diarrhea	2,888	197 (31%)	573 (25%)	0.004
Heart disease	2,875	39 (6.2%)	218 (9.7%)	0.008
Cerebrovascular disease	2,875	30 (4.8%)	165 (7.3%)	0.027
Neurologic disease	2,875	10 (1.6%)	41 (1.8%)	0.714
Lung disease	2,875	50 (8.0%)	201 (8.9%)	0.473
Rheumatic disease	2,875	21 (3.4%)	81 (3.6%)	0.781
Gastrointestinal disease	2,875	6 (1.0%)	18 (0.8%)	0.694
Liver disease	2,875	45 (7.2%)	118 (5.2%)	0.060
Diabetes	2,875	89 (14%)	361 (16%)	0.280
Renal disease	2,875	134 (21%)	538 (24%)	0.205
Cancer/immunological disease	2,875	239 (38%)	834 (37%)	0.567
Regular contact to pets of others	2,887	34 (5.4%)	124 (5.5%)	>0.9
Ownership of pets other than dogs/cats	2,890	43 (6.9%)	72 (3.2%)	<0.001
Professional contact to livestock or pets	2,881	91 (15%)	72 (3.2%)	<0.001
