# Supplementary material for: The transmission risk of multidrug-resistant organisms between hospital patients and their pets – a case−control study, Germany, 2019 to 2022
Source: Euro Surveill. 2024 Sep 26;29(39):2300714. doi: 10.2807/1560-7917.ES.2024.29.39.2300714 (PMC11484343; doi:10.2807/1560-7917.ES.2024.29.39.2300714)
Supplement: Supplement1 [file 23-00714_HACKMANN_Supplement1_StudyProtocol.pdf]

CHARITÉ

CharitéCentrum für diagnostische und präventive Labormedizin

Institut für Hygiene und Umweltmedizin,
Hindenburgdamm 27, 12203 Berlin
Direktorin: Prof. Dr. med. Petra Gastmeier

Nationales Referenzzentrum für Surveillance von nosokomialen Infektionen

AMR-Pet

Antimicrobial–resistant pathogens transmitted via pets

Study Protocol

Principal investigators:	Prof. Dr. Petra Gastmeier, Charité Berlin
	PD Dr. Rasmus Leistner, Charité Berlin
Version:	1.4
Funding source:	Bundesministerium für Gesundheit (BMG)
DRKS Study ID:	DRKS00030009

Table of Contents

1. Synopsis 3
2. Persons/institutions involved in the study 5
3. Background 6
4. Objectives and hypotheses 6
5. Methods 7
6. Data collection 10
7. Data management 10
8. Statistical analysis 10
9. Ethics 12
10. Publication of research findings 12
11. Appendix 13

List of Abbreviations

3GCR-GNB	3rd Generation Cephalosporin resistant Gram-negative bacteria
95%-CI	95% confidence interval
CR-GNB	Carbapeneme resistant Gram-negative bacteria
DAG	Directed acyclic graph
ESBL	Extended spectrum beta lactamase
ICU	Intensive care unit
MDR-GNB	Multidrug resistant Gram-negative bacteria
MDRO	Multidrug resistant organism
MRSA	Methicillin resistant *Staphylococcus aureus*
OR	Odds Ratio
VRE	Vancomycin resistant Enterococcus
WGS	Whole Genome Sequencing

1. Synopsis

Title of Protocol	Transmission of antimicrobial-resistant pathogens between hospital patients and their owners
Protocol Chair	Prof. Dr. Petra Gastmeier
Objectives<br>Primary Objective<br><br>Secondary Objectives	 To determine the relevance of pet ownership as a risk factor for MDRO colonization in hospital patients on admission.<br>To determine the rate of transmission between pets and owners via WGS.<br>To determine the prevalence of MDRO colonization in the included pets.
Hypotheses/Outcome<br>Primary hypothesis and outcome<br><br>Secondary hypotheses and outcomes	 If pets are a relevant risk factor for MDRO colonization in their owners, the rate of pet ownership will be higher in MDRO-Positives than in MDRO-Negatives. The primary outcome is the odds ratio of pet ownership in MDRO-positive hospital patients and MDRO-negative hospital patients. MDRO colonization is defined as recovery of MDRO isolates (MRSA, VRE, 3GCR-GNB or CR-GNB) <3 days after hospital admission.<br>Approximately 20% of pets will be tested MDRO-positive and will be available for comparison of MDRO isolates of pet and owner. Outcomes are the rate of MDRO co-carriage among all tested pet-owner-pairs based on WGS analyses as well as the prevalence of MDRO colonization in pets.
Study Design	The study is a prospective case control study, which will be performed at three hospitals of the Charité – University Hospital in Berlin. Cases are defined as patients who test positive for MDROs in swab samples from nose and rectum within 3 days after hospital admission. Controls are defined as patients who test negative for MDROs during the first 3 days of their hospital stay. Retrospective matching can be performed based on the ward.

Study Population<br>Inclusion Criteria<br><br>Exclusion Criteria	 Adult (min. 18 years) inpatients from non-ICU wards during the first 3 days of their hospital stay, with written informed consent.<br>Age <18 years, patient under legal guardianship, language barrier (patient not speaking German, English or Turkish), patient unable to answer questions due to poor general health
Sample Size	Assuming 50% of MDRO-negative and 55% of MDRO-positive patients are pet owners and a case-control-ratio of 1:2, 1.226 cases and 2.452 controls will have a power of at least 80% to detect a least extreme odds ratio of 1.22 with a two-sided confidence level of 95%.
Data Analyses	For statistical analyses we calculate the OR and their 95%-CI for pet ownership and other risk factors for MDRO-carriage in hospital patients. We will perform univariable and multivariable analyses. Multivariable analysis models will be based on previously designed DAGs.

2. Persons/institutions involved in the study

Sponsor

The study is funded by the German Federal Ministry of Health (BMG) under the grant number ZMVI1-2518FSB704.

Study Coordinator

Prof. Dr. Petra Gastmeier
PD Dr. Rasmus Leistner
Dr. Carolin Hackmann

Institute of Hygiene and Environmental Medicine
Charité – Universitätsmedizin Berlin
Hindenburgdamm 27
12203 Berlin

Associated partners and Institutions

Prof. Dr. Stefan Schwarz
Dr. Antina Lübke-Becker
Institute of Microbiology and Epizootics
Department of Veterinary Medicine
Freie Universität Berlin
Robert-von-Ostertag-Str. 7
14163 Berlin

3. Background

The majority of nosocomial infections are endogenous and therefore brought to the hospital by the patients themselves. The microbiome of every human depends on multiple factors, including the living organisms around him or her. Pets can play a crucial role in the transmission of microbes due to the close contact between pets and owners in most households. In Germany, about 60% of people own at least on pet, 50% of them even more than one. The most common pets are cats, dogs, small animals and birds.

Transmission of MDROs between pets and owners has been demonstrated in previous studies. Transmission can comprise the whole pathogen as well as mobile genetic elements like plasmids. This way, antibiotic resistance can be transmitted between pathogens of the same or distinct species. MDROs transmitted most frequently between pets and owners are Methicillin-resistant *Staphylococcus pseudintermedius*, Vancomycin-resistant Enterococci and ESBL- or Carbapenemase-producing Gram-negative bacteria.

Literature on the transmission of MDROs from pets to humans is scarce and mostly refers to Methicillin-resistant Staphylococcus aureus. Transmission of ESBL-producing Enterobacterales or VRE is mostly based on veterinary screening surveys and anecdotal reports of household transmissions.

The zoonotic component of MDRO infection and colonization is insufficiently investigated at this point. There is a need for high quality epidemiologic studies paired with state-of-the-art genomic analyses to gain more insights in this topic.

The most important MDROs causing nosocomial infections in Germany are MRSA, VRE and MDR-GNB. This study will therefore focus on these three groups of pathogens.

4. Objectives and hypotheses

Primary Objective and Hypothesis

- To determine the relevance of pet ownership as a risk factor for MDRO colonization in hospital patients on admission.
- If pets are a relevant risk factor for MDRO colonization in their owners, the rate of pet ownership will be higher in MDRO-Positives than in MDRO-Negatives.

Secondary Hypotheses and Objectives

- To determine the rate of transmission between pets and owners via WGS.
- To determine the prevalence of MDRO colonization in the included pets.
- Approximately 20% of pets will be tested MDRO-positive and will be available for comparison of MDRO isolates of pet and owner.

5. Methods

Study Design

The study is a prospective case control study, performed at three hospital sites of the Charité University hospital in Berlin: Campus Mitte, Campus Virchow-Klinikum and Campus Benjamin Franklin. Cases are defined as patients who test positive for MDROs the swab samples from nose and rectum within 3 days after hospital admission. Controls are defined as patients who test negative for MDROs during the first 3 days of their hospital stay. Participants are selected from the Charité patient population. To detect potential cases, we approach patients who tested positive for MDROs in the 72 hours after hospital admission and patients who were infected or colonized with MDROs in prior hospital stays at the Charité. To detect potential controls, we approach patients who tested negative for MDROs in the 72 hours after hospital admission and newly admitted patients during the first 72 hours of their hospital stay. We aim at a case-control ratio of 1:2.

All participants complete a questionnaire-based interview performed by our study staff members. It includes information on age, sex, body mass index (height, weight), living situation (alone, in a nursing home or shared flat/family) and diet (vegan, vegetarian, mixed diet). Additionally, it includes questions on well known risk factors for MDRO acquisition: prior hospitalization, antibiotic intake, travelling habits, prior detection of MDROs, presence of urinary tract catheters or intravenous catheters, underlying diseases (diarrhea, chronic kidney failure, diabetes, cancer) and contact to domesticated animals (cows, pigs, poultry, horses). To measure underlying co-morbidities, we obtain the Charlson Comorbidity Index (CCI) for each participant. All participants are questioned whether they have contact to pets in their profession, own pets (including number of pets and species) or have regular contact to pets of other (>3 hours per week). If the participants own cats or dogs, they are asked to answer further questions regarding their pet's health status in the past 6 months (diarrhea, urinary tract infections, diabetes, skin infections, invasive procedures, antibiotic intake, other self-applied treatments). In addition, they are asked about the diet of their pets (canned pet food,

dry pet food, raw meat, leftovers), the closeness of contact (pets sleep in bed, lick hands or face, are washed in the shower/bathtub) and behavior of the pets (vomiting in the house, urination or defecation in the house, time spend outside of the house). They are further asked for how long they have had pets and about their hand hygiene after touching pets.

Nasal and rectal swabs of all participants are tested for MRSA, VRE, 3GCRE and CRE (see Microbiological Methods for further information). All owners of cats or dogs (further referred to as pet owners) receive screening kits as soon as they are discharged from the hospital to test their pets at home. If there is no response, two reminders are sent at an interval of two weeks, respectively. Pet owners collect swab samples of their pet's throat and stool and send them back to our institute in prepared envelopes. The samples are analyzed the same way as the human samples. In addition to the swab samples, pet owners answer an additional short questionnaire to provide information on the health status of each pet at the time of the sampling (age, sex, current diseases and antibiotic intake). When pathogens isolated from human and pet samples from the same household are phenotypically identical, we perform Whole Genome Sequencing (WGS) analysis to examine genetic relatedness (see Microbiological Methods for further information). Participants are informed about the outcome of the microbiological analyses of their own and their pet's samples.

Study Population

Participants in this study were adult inpatients (min. 18 years) from non-ICU wards during the first 3 days of their hospital stay, with written informed consent.

Excluded were patients under the age of 18 years, patients under legal guardianship, patients with a language barrier (patient not speaking German, English or Turkish) and patients unable to answer questions due to poor general health.

Outcomes

The primary outcome is the odds ratio of pet ownership in MDRO-positive hospital patients and MDRO-negative hospital patients. MDRO colonization is defined as recovery of MDRO isolates (MRSA, VRE, 3GCR-GNB or CR-GNB) <3 days after hospital admission.

Secondary outcomes are the rate of MDRO co-carriage among all tested pet-owner-pairs based on WGS analyses as well as the prevalence of MDRO colonization in pets.

Microbiological Methods

Nasal swab samples

Nasal and throat swab samples of humans and pets will be inoculated onto chrome MRSA agar (Chrom MRSA, bioMérieux, Marcy-l'Étoile, France), for 24 hours at 37°C. For the confirmation of *S. aureus*, the latex agglutination tests detecting clumping factor (Staphaurex Plus, Remel, Lenexa, USA) will be used. For confirmation of Methicillin resistance, the penicillin-binding-protein latex agglutination test (PBP2 Test Kit, Oxoid, Wesel, Germany) will be used. If necessary, antimicrobial susceptibility testing will be performed with Vitek®2 System (bioMérieux, Marcy-l'Étoile, France).

Rectal and stool swab samples

Rectal swabs and stool swab samples of humans and pets will be inoculated onto chrome agar (ChromID ESBL-Agar, bioMérieux, Marcy-l'Étoile, France) for the identification of ESBL-positive isolates and McConkey agar plates (McConkey-Agar, bioMérieux, Marcy-l'Étoile, France) for selection of GNB for 24 hours at 37°C. Carbapeneme, Chinolone and Penicilline resistance will be identified via agar diffusion using Imipenem, Meropenem, Ertapenem, Ciprofloxacin and Piperacillin antibiotic plates. Identification of MDR-GNB species and antimicrobial susceptibility testing will be performed with Vitek®2 System (bioMérieux, Marcy-l'Étoile, France). Results will be interpreted in concordance with the European Committee on Antimicrobial Susceptibility Testing definitions (EUCAST, http://www.eucast.org).

For the identification of VRE, incubation on ChromID® VRE agar plates (bioMérieux, Marcy-l'Étoile, France) for 48 hours at 36±1°C will be performed. For further analysis the disc diffusion method with 5 µg vancomycin and 30 µg teicoplanin from MASTDISCS® (Mast Group Ltd., Bottle, United Kingdom) will be used. Plates will be incubated for 24 hours at 37°C. Identification of species and antimicrobial susceptibility testing will be performed with Vitek®2 System (bioMérieux, Marcy-l'Étoile, France). Results will be interpreted in concordance with the European Committee on Antimicrobial Susceptibility Testing definitions (EUCAST, http://www.eucast.org).

Whole Genome Sequencing

All resistant isolates will be stored as cryocultures at -80°C until further sequencing analysis. Subcultures will be cultured on blood agar and incubated overnight at 37°C. DNA extraction will be performed using UltraClean Microbial DNA isolation kit (Qiagen, Hilden, Germany) following the manufacturer's instructions. DNA quantity and purity will be measured by QuantiFluor ONE dsDNA System (Promega GmbH, Mannheim, Germany) and Eppendorf Biophotometer (Eppendorf AG, Hamburg, Germany). Short read sequencing will be performed on the MiSeq system (Illumina Inc., San Diego, USA) according to the manufacturer's instructions. For generating short read sequencing libraries from genomic DNA the Nextera XT DNA library preparation kit (Illumina Inc. San Diego, USA) will be used. De novo assembly with the Velvet assembler and gene-by-gene comparison approach will be performed using the SeqSphere+ software version 1.4.9 (Ridom GmbH, Muenster, Germany). For gene-by.-gene comparison published cgMLST task templates will be used for each type of pathogen.

6. Data collection

Data collection will be performed by study personnel on the respective ward (at bed-side). Information collected from each patient can be seen in the attached questionnaire (see Appendix).

Data from pets (swab samples, questionnaires on pets health status) will be provided by the pet owners.

7. Data management

Study personnel will collect a written declaration of informed consent on paper forms, which will be stored securely at the study site. Medical data will be collected in electronic case report forms (eCRFs) using mobile notebooks and will be transferred to and stored on a local secured server. Only study personnel will be able to access the collected data.

8. Statistical analysis

Sample size

Sample size calculation was performed in OpenEpi, Version 3, based on the following assumptions:

Assuming the rate of pet ownership to be 50% in MDRO-Negatives and 55% in MDRO-Positives, a power of 80%, alpha=0.05 and a case-control ratio of 1:2, at least 1,226 cases and 2,452 controls are needed. The total sample size would be 3,678 patients.

Recruitment of patients will be conducted in three Charité hospitals, the total number of newly admitted patients during the study period is estimated to be about 250,000. An annual number of 3,000 patients with a MDRO colonization (2,200 MDR-GNB, 500 MRSA, 300 VRE) detected during the first 3 days after hospital admission is assumed. Based on experience, a dropout rate of 50% during recruitment is assumed. An additional dropout rate of 50% is assumed for the screening of pets by owners. The rate of MDRO-positive pets is estimated at a maximum of 20%. Based on these figures, during a study period of 2 years, 3,000 cases and 3,000 controls should be recruited for the case-control comparison. Around 150 pairs of MDRO-positive pets and owners should be assessed for transmission of MDROs via WGS.

Statistical plan

Description of parameters will be displayed as figures and percentages for categorical parameters and median and interquartile range (25% percentile, 75% percentile) for continuous parameters. Depending on the distribution of the parameters, differences will be tested using Fisher's Exact test, Chi-square test, T-test or Wilcoxon rank-sum test.

The rate of pet ownership will be compared between MDRO-positive and MDRO-negative patients as well as between the groups of MRSA-positives and negatives, VRE-positives and negatives and MDR-GNB-positives and negatives. If applicable retrospective matching based on the admission ward will be performed to analyze the three groups. In univariable and multivariable analyses, crude and adjusted odds ratios and 95% confidence intervals will be calculated. Logistic regression models will be used. Multivariable models will be based on beforehand generated DAGs, using the software DAGitty (http://www.dagitty.net/dags.html).

The rate of transmission between pets and owners will be calculated as the percentage of pet-owner co-carriage out of all pet-owner pairs.

The rate of MDRO-positive pets will be calculated as a prevalence (number of MDRO-positive pets/all tested pets).

9. Ethics

Ethical approvement

The study was approved by the Charité University Medicine ethics committee (approval number EA4/042/19).

Informed consent

Prior to participation in this study, each patient is given detailed information about the study, its risks and benefits as well as his or her rights of withdrawal. Participation in the study is possible only after signing a written declaration of informed consent.

Confidentiality

Data collected in this study will be processed pseudonymized. Information linking patients medical data to personal data will be stored in a secure location at the site. Personal data will be used exclusively to inform patients on their status of colonization. When results from this study are published, the individuals identity will remain confidential.

10. Publication of research findings

Results from this project will be published in scientific journals. Interim results will be presented throughout the study period at national and international congresses as well as meetings of research networks.

11. Appendix

Questionnaire (paper form)

Pat.-ID

CHARITÉ

CharitéCentrum für diagnostische und präventive Labormedizin

Institut für Hygiene und Umweltmedizin,
Hindenburgdamm 27, 12203 Berlin
Direktorin: Prof. Dr. med. Petra Gastmeier

Nationales Referenzzentrum für Surveillance von nosokomialen Infektionen

Questionnaire for the Study
"The Transmission of Antibiotic-Resistant Pathogens by House Pets"

**Patient:**	❑ Case	❑ Control	Date:____________
**Pathogen*:**	MRSA:_________	VRE:__________	MRGN:__________
Location	_______________	_______________	_______________
Species		_______________	_______________
Attribute			_______________
**Screening:**	❑ MRSA	❑ VRE	❑ MRGN

**0 = not tested, 1 = tested negative, 2 = tested positive*

Sex: ❑ Male ❑ Female ❑ Third

Age: _____ years old **Height:** _____ cm **Weight:** _____ kg

1. **What has your diet been during the past six months?**
   ❑ Vegan
   ❑ Vegetarian (dairy products, eggs)
   ❑ Mixed diet (fish and meat)

2. **What kind of household have you lived in during the past six months?**
   ❑ Alone
   ❑ In a nursing home
   ❑ In a shared flat / with a family

3. **Have you been hospitalized during the past six months (incl. in-patient rehab stays), not including your present hospital stay?**
   ❑ Yes ❑ No ❑ I don't know

4. **Have you taken antibiotics during the past six months?**
   ❑ Yes, namely ____________________ ❑ No ❑ I don't know

03_Fragebogen_AMRPet_18_06_23 ENGLISH.doc

CHARITÉ - UNIVERSITÄTSMEDIZIN BERLIN

Seite 1 von 5

5. **Have you traveled abroad during the past six months?**
   - ❑ Yes, in Europe — Country: ____________________
   - ❑ Yes, to Asia — Country: ____________________
   - ❑ Yes, to South/Central America/ The Caribbean — Country: ____________________
   - ❑ Yes, to North America — Country: ____________________
   - ❑ Yes, to Africa — Country: ____________________
   - ❑ Yes, to Australia/New Zealand — Country: ____________________
   - ❑ No

6. **Do you have a urinary tract catheter (UTC) or an intravenous catheter (IVC)?**
   ❑ Yes, UTC ❑ Yes, IVC ❑ No

7. **Were you diagnosed for any of the following pathogens during the past six months?**
   - ❑ Yes, ❑MRSA ❑VRE ❑MRGN
   - ❑ No
   - ❑ I don't know

8. **Have you had diarrhea during the pasts six months?**
   ❑ Yes ❑ No ❑ I don't know

9. **Do you have one of the following diseases?**

Diabetes mellitus	❑ Yes	❑ No
Chronic kidney failure	❑ Yes	❑ No
Cancer or leukemia	❑ Yes	❑ No

10. **Have you been in direct contact with domesticated animals (horses, pigs, cows, poultry, etc.) during the last six months?**
    ❑ Yes, with ____________________ ❑ No ❑ I don't know

11. **Do you have contact with animals in your job?**
    - ❑ Yes, with ____
    - ❑ No

12. **Do you have pets?**
    - ❑ Number of dogs: __________
    - ❑ Number of cats: __________
    - ❑ Others: ____________________________
    - ❑ No

13. **Do you have contact with other people's pets (for more than three hours per week)?**

    ____

    ____

If you do not have a dog or cat of your own, you are now finished with the questionnaire.

Additional questions for owners of dogs and/or cats:

14. How long have you had pets?

Dog
- ❑ Less than 1 year
- ❑1 – 5 years
- ❑ 5 – 10 years
- ❑ More than 10 years

Cat
- ❑ Less than 1 year
- ❑1 – 5 years
- ❑ 5 – 10 years
- ❑ more than 10 years

15. Has your pet had diarrhea during the past six months?

Dog
- ❑ Yes
- ❑ No
- ❑ I don't know

Cat
- ❑ Yes
- ❑ No
- ❑ I don't know

16. Has your pet had a urinary infection during the past six months?

Dog
- ❑ Yes
- ❑ No
- ❑ I don't know

Cat
- ❑ Yes
- ❑ No
- ❑ I don't know

17. Does your pet have diabetes?

Dog
- ❑ Yes
- ❑ No
- ❑ I don't know

Cat
- ❑ Yes
- ❑ No
- ❑ I don't know

18. Has your pet suffered from a skin disease (including ear infections) during the past six months?

Dog
- ❑ Yes
- ❑ No
- ❑ I don't know

Cat
- ❑ Yes
- ❑ No
- ❑ I don't know

19. Has your pet undergone an invasive procedure (e.g. surgery) during the past six months?

Dog
- ❑ Yes, ________________________
  ____
- ❑ No
- ❑ I don't know

Cat
- ❑ Yes, ________________________
  ____
- ❑ No
- ❑ I don't know

20. **Has your pet been treated with antibiotics (e.g. for diarrhea or respiratory problems) during the past six months?**

Dog	*Cat*
❑ Yes, with ________________	❑ Yes, with ________________
❑ No	❑ No
❑ I don't know	❑ I don't know

21. **Have you treated your pet yourself (e.g. applying ointments) during the past six months?**

Dog	*Cat*
❑ Yes, ________________ ________________	❑ Yes, ________________ ________________
❑ No	❑ No
❑ I don't know	❑ I don't know

22. **What kind of food does your pet eat?**

Dog	*Cat*
❑ Canned pet food	❑ Canned pet food
❑ Dry pet food	❑ Dry pet food
❑ Raw meat	❑ Raw meat
❑ Leftovers	❑ Leftovers
❑ Other	❑ Other

23. **Do you have any of the following types of contact with your pet?**

Your dog...	*Your cat...*
❑ sleeps in your bed	❑ sleeps in your bed
❑ licks your hands	❑ licks your hands
❑ licks your face	❑ licks your face
❑ is washed in the shower/bathtub	❑ is washed in the shower/bathtub

Mark the appropriate position on the scale.

24. How often has your pet vomited in your home in the last six months?

Dog

never — seldom — often — always

Cat

never — seldom — often — always

25. How often does your pet urinate or defecate in your home?

Dog

never — seldom — often — always

Cat

never — seldom — often — always

26. How much time do your pets spend outside?

Dog

never — seldom — often — always

Cat

never — seldom — often — always

27. How regularly do you wash your hands after petting your pet?

Dog

never — seldom — often — always

Cat

never — seldom — often — always

Thank you for participating!
